# Comparison of Global DNA Methylation Patterns in Human Melanoma Tissues and Their Derivative Cell Lines

**DOI:** 10.3390/cancers13092123

**Published:** 2021-04-28

**Authors:** Euan J. Rodger, Suzan N. Almomani, Jackie L. Ludgate, Peter A. Stockwell, Bruce C. Baguley, Michael R. Eccles, Aniruddha Chatterjee

**Affiliations:** 1 Department of Pathology, Otago Medical School—Dunedin Campus, University of Otago, Dunedin 9054, New Zealand; suzan.almomani@otago.ac.nz (S.N.A.); jackie.ludgate@otago.ac.nz (J.L.L.); peter.stockwell@otago.ac.nz (P.A.S.); 2 Maurice Wilkins Centre for Molecular Biodiscovery, Auckland 1010, New Zealand; b.baguley@auckland.ac.nz

**Keywords:** methylation, cell culture, epigenetics, cancer, in vitro

## Abstract

**Simple Summary:**

Cancer cell lines are a defined population of cells, originally sourced from tumour tissue, that can be maintained in culture for an extended period of time. They are a critical laboratory-based model, and are frequently used to unravel mechanisms of cancer cell biology. In all cells, gene activity is in part regulated by DNA methylation, the epigenetic process by which methyl groups are added to DNA. In this study, we demonstrate that at a global level, DNA methylation profiles are globally well conserved, but we identify specific sites that are consistently more methylated in tumour-derived cell lines compared to the original tumour tissue. The genes associated with these common differentially methylated regions are involved in important cellular processes and are strongly enriched for epigenetic mechanisms associated with suppression of gene activity. This study provides a valuable resource for identifying false positives due to cell culture and for better interpretation of future cancer epigenetics studies.

**Abstract:**

DNA methylation is a heritable epigenetic mark that is fundamental to mammalian development. Aberrant DNA methylation is an epigenetic hallmark of cancer cells. Cell lines are a critical in vitro model and very widely used to unravel mechanisms of cancer cell biology. However, limited data are available to assess whether DNA methylation patterns in tissues are retained when cell lines are established. Here, we provide the first genome-scale sequencing-based methylation map of metastatic melanoma tumour tissues and their derivative cell lines. We show that DNA methylation profiles are globally conserved in vitro compared to the tumour tissue of origin. However, we identify sites that are consistently hypermethylated in cell lines compared to their tumour tissue of origin. The genes associated with these common differentially methylated regions are involved in cell metabolism, cell cycle and apoptosis and are also strongly enriched for the H3K27me3 histone mark and PRC2 complex-related genes. Our data indicate that although global methylation patterns are similar between tissues and cell lines, there are site-specific epigenomic differences that could potentially impact gene expression. Our work provides a valuable resource for identifying false positives due to cell culture and for better interpretation of cancer epigenetics studies in the future.

## 1. Introduction

DNA methylation is a stable and somatically heritable epigenetic modification. In mammals, DNA methylation involves attachment of a methyl group to a cytosine, usually in the context of a CpG dinucleotide. Methylation patterns are established during early embryogenesis and play a key role in cellular differentiation, lineage commitment and maintenance of cell identity [1,2]. In somatic cells, DNA methylation patterns are established on the newly synthesized DNA strand in daughter cells by DNA methyltransferase 1 (DNMT1). Therefore, DNA methylation provides a mechanism for the heritability of epigenetic information [3]. Aberrant DNA methylation is now established as a major feature of cancer and plays a major role in the function of tumour cells [4]. Immortalized cancer cell lines are often used in place of primary cells to study the underlying processes of epigenetic events and epigenetic mechanisms in tumour cells and biomarker validation through functional studies and drug discovery [5]. For example, cell lines provided an excellent pre-preclinical model for the discovery and development of the FDA-approved epigenetic drugs, such as the demethylating agents and histone-modifying agents, and continue to be widely used for functional studies and development of epigenetic drugs [6,7,8]. More recently, the ability to edit specific DNA methylation marks using CRISPR-based systems is providing a new way to investigate causal effects of methylation changes in cancer [9,10]. Again, cell lines provide the ideal system to develop these methods to understand cancer cell function [11,12,13]. A bulk tumour includes a diverse range of cells (including the presence of non-tumour cells) and this heterogeneity is likely to result in a non-uniform distribution of genetically and epigenetically distinct subpopulations of tumour cells [14]. Cell lines provide a relatively homogeneous population of cells compared to the heterogeneous tumour tissue samples, making them less confounded for discovering and confirming mechanisms related to the cancer methylome in some contexts [15].

Although cancer cell lines have been an indispensable and a very widely used model for cancer biology studies for decades, they often do not fully represent the epigenetic state that exists in vivo [16,17,18]. It is well documented that cell lines undergo genetic and epigenetic changes during initial establishment [19,20]. This warrants investigation of the similarities and differences in DNA methylation patterns between tumour tissues and their derived cell lines, thus providing better interpretation of cancer epigenetics. Several studies attempting to address this question have profiled epigenetic features in large panels of cell lines and tissues [21,22,23,24,25]. Although these studies have provided useful information and facilitated the understanding of epigenetic differences that arise due to cell culture, a major limitation is that they have analysed a group of cell lines and an unmatched series of tissues instead of tumour tissues with matching cell lines derived from them. Without the direct profiling of tissues and the cell lines derived from those tissues, it will not be possible to understand epigenetic differences and the associated consequences due to establishment of culture from tissues. The availability of tumour tissues and their matching derived cell lines is rare; to our knowledge, only limited epigenetic data are available for such matched samples.

Here, we provide the first genome-scale methylation profiles of metastatic melanoma tumour tissues and the corresponding cell lines derived from these tissues using reduced representation bisulfite sequencing (RRBS) [26]. RRBS is a widely used and reproducible technique for the investigation of genome-scale DNA methylation patterns at single-nucleotide resolution [27,28]. Although size selection of MspI-digested fragments results in ~2.3% coverage of the human genome, RRBS significantly enriches for CpG sites (5.7-fold) [29] and CpG islands (~30-fold) [28]. We present a comprehensive comparison of DNA methylation profiles from melanoma cell lines and their corresponding tumour tissues. We identify common site-specific methylation differences between cell lines and corresponding tumour tissues, and investigate the genomic features of these sites. We provide valuable data and resources that will assist in the investigation of cell lines as a model of cancer epigenetics studies, and critically consider the site-specific differences that arise due to cell culture as a potential confounder for future studies.

## 2. Results

### 2.1. Generating RRBS Methylomes from Melanoma Tumour Tissue and Matched Cell Lines

RRBS was used to establish genome-wide DNA methylation maps of melanoma tissues (53T and 60T) and their derived cell lines (53C and 60C, respectively), producing a total of 101.4 million 100 bp sequenced reads that were then mapped to the GRCh37 reference human genome (Supplementary Table S1). We analysed DNA methylation patterns using MspI fragments as the unit of analysis as previously described [30,31]. We obtained high-quality information (fragments having 10 or more reads at ≥2 CpG sites) that covered 707,368, 541,502, 974,929 and 1,458,233 CpG sites in 53T, 53C, 60T and 60C, respectively (Supplementary Table S2).

### 2.2. Metastatic Cell Lines Globally Maintain Tissue Methylation Patterns and Follow Their Origin

**Figure 1:**
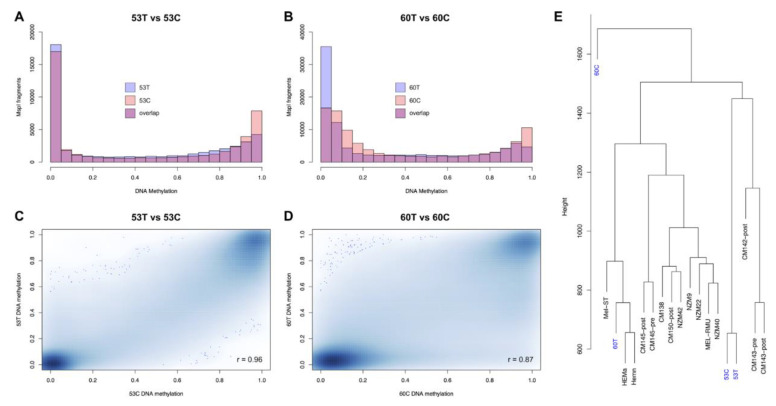
Global methylation patterns in melanoma tissues and derived cell lines. (**A**,**B**) Histograms showing the degree of methylation in all analysed RRBS fragments of 53T compared to 53C (**A**) and 60T compared to 60C (**B**). The *x* axis represents methylation level on a 0–1 scale and the *y* axis is the number of RRBS fragments in each bin. (**C**,**D**) Smoothed colour density scatterplots show the correlation of methylation in tissues (*y* axis) with respect to their derived cell lines (*x* axis). (**E**) Hierarchical clustering of RRBS fragments together with 3 normal melanocyte cell lines (Mel-ST, HEMa-LP and HEMn-LP) and 12 metastatic melanoma cell lines (CM145-post, CM145-pre, CM150-post, CM142-post, CM138, CM143-pre, CM143-post, MEL-RMU, NZM9, NZM22, NZM40 and NZM42).

As expected from previous analyses of human cell lines and somatic tissues [22], the methylation levels in all samples showed a bimodal distribution. Tumour samples had a higher proportion of fully unmethylated fragments, whereas the cell line samples had a higher proportion of fully methylated fragments (Figure 1A,B). We found that global DNA methylation levels remained relatively conserved between the tumours and their corresponding cell cultures for both 53T vs. 53C (Pearson’s *r* = 0.96, Figure 1C) and 60T vs. 60C (*r* = 0.87, Figure 1D, Supplementary Table S3). To compare the global RRBS methylome patterns in these tumours and the corresponding cell lines in a broader context, we utilised RRBS data from three normal melanocyte and 12 metastatic melanoma cell lines that were generated and analysed using the same RRBS methods and protocols [32,33]. Hierarchical clustering showed clear separation of melanoma tumour cell lines from the normal melanocyte cell lines (Mel-ST, HEMn-LP and HEMa-LP), further indicating a tumour-specific global methylation signature in melanoma cell lines compared to normal cell lines (Figure 1E). The 53C and 53T methylomes clustered together in the same branch indicating conserved global methylation between the tumour and corresponding cell line. For the 60C and 60T samples, although they clustered together in a main branch, this branch also included the normal melanocytes cell lines. Investigation of the median global methylation values revealed that 60C and 60T are both substantially hypomethylated (median global methylation 0.11 and 0.21, respectively, Supplementary Table S4) compared to the other melanoma cell lines. As a result, these two methylomes clustered together with the normal melanocyte cell lines, which are also substantially hypomethylated. These results provide strong evidence that the global DNA methylation profiles are broadly preserved between cell lines and their tumour tissue of origin.

### 2.3. RRBS Methylomes of Melanoma Cell Lines Are Relatively Hypermethylated, When Compared to Their Corresponding Tumour Tissues

**Figure 2:**
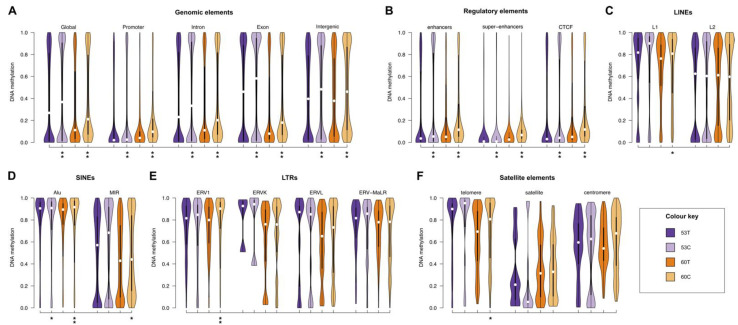
Element-wise methylation profiles in melanoma tissues and derived cell lines. (**A**–**F**) Equal area violin plots of 53T, 53C (purple), 60T and 60C (orange) show the distribution of methylation in different genomic elements. (**A**) Gene promoters (−2 kb to +1 kb from the TSS), introns, exons and intergenic elements (>2 kb upstream from the nearest TSS). (**B**) Regulatory elements: enhancers, super-enhancers and CTCF binding sites (**C**) LINEs: L1 and L2. (**D**) SINEs: Alu and MIR. (**E**) LTRs: ERV1, ERVK, ERVL and ERV-MaLR. (**F**) Satellite elements: telomere, satellite, centromere. In all cases, the y axis represents methylation level on a 0–1 scale. * *p* < 0.05, ** *p* < 1 × 10^−10^ (tissue vs. cell line, Wilcoxon rank sum test).

We observed that both the cell lines were globally hypermethylated as compared to their corresponding tumour tissue. In both cases, we identified 10% higher median methylation in cell lines compared to tumour tissues (53T and 53C median methylation was 0.27 vs. 0.37, respectively, while for 60T and 60C the median methylation was 0.11 vs. 0.21, respectively (Supplementary Table S4). As expected, for both cell lines and tumour tissues, gene promoter regions (defined as −2 kb to +1 kb from the transcription start site) were generally unmethylated (median methylation = 0.05), but gene bodies and intergenic regions were methylated (0.27 and 0.43, respectively). The pattern of increased methylation in the cell lines was apparent in gene bodies (~10%) and intergenic regions (~10%; Figure 2A, Supplementary Table S4). Enhancers, super-enhancers and CTCF binding sites were generally unmethylated (median methylation = 0.06, 0.03 and 0.06, respectively) for both cell lines and tissues. The increased methylation pattern in cell lines compared to the tumour tissue of origin was observed in all of these elements (~5%; Figure 2B). Overall, repeat elements also showed increased methylation in both melanoma cell lines compared to tumour tissues, but only by a small amount (<4%) for most elements. In 60C, telomeres and ERV1 elements were more methylated by ~10% (Figure 2C–F).

### 2.4. Melanoma Cell Lines Demonstrate Region-Specific Methylation Differences in Comparison to the Tumour Tissues from Which They Were Derived

**Figure 3:**
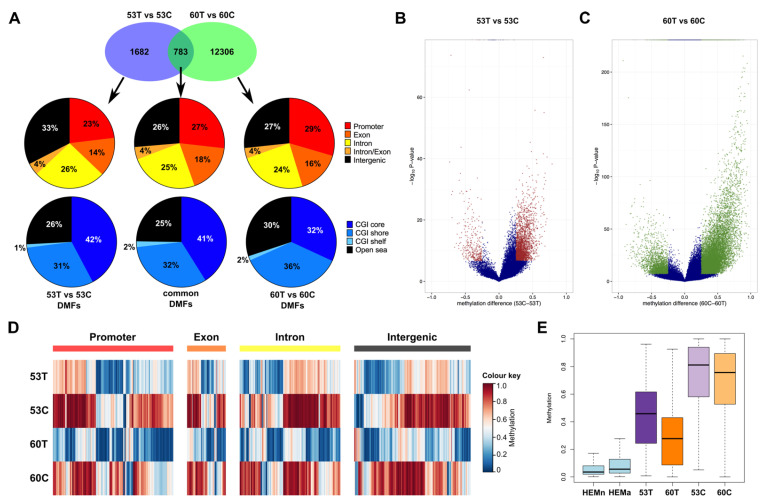
Differential methylation patterns in melanoma tissues and derived cell lines. (**A**) The Venn diagram shows the number of significantly differentially methylated fragments (DMFs) in each comparison (Fisher’s exact test; Bonferroni-adjusted *p* < 0.01, methylation difference ≥25%) and the 783 that were in common. The pie charts show the proportion of DMFs overlapping: (top) gene promoters (−2 kb to +1 kb from the TSS), exons, introns, intron/exon junctions and intergenic elements (>2 kb upstream from the nearest TSS); and (bottom) CpG island (CGI) cores, shores, shelfs and open sea. (**B**,**C**) Volcano plots showing the methylation changes of tissue vs. cell line. The *x* axis shows methylation differences of the analysed fragments and the *y* axis shows the −log10 of the *p*-values. Blue data points indicate fragments that were not significant and DMFs are shown in red (for 53T vs. 53C) or green (for 60T vs. 60C). (**D**) Methylation heatmaps of all common DMFs in different genomic elements (blue = unmethylated, red = fully methylated). (**E**) Boxplots show the methylation distribution of DMFs in normal melanocyte cell lines (light blue), 53T, 53C (purple), 60T and 60C (orange).

The differences in methylation patterns observed in different genomic elements led us to interrogate region-specific methylation differences. We identified 2465 differentially methylated fragments (DMFs) in the comparison between 53T and 53C and 13,089 DMFs in the comparison between 60T and 60C (Figure 3A, Supplementary Tables S5 and S6). All DMFs had a Bonferroni-adjusted significance level <0.01 and methylation difference ≥25%. Of the 783 DMFs in common between the two comparisons, 27% were in gene promoters, 47% in gene bodies and 26% in intergenic regions (Figure 3A,D, Supplementary Table S7). Compared to all analysed RRBS fragments, there was a higher proportion of DMFs in intergenic regions (Supplementary Figure S1). The 783 DMFs were also distributed across different CGI features—cores (41%), shores (32%), shelfs (2%) and open sea (25%) regions (Figure 3A, Supplementary Table S7).

Consistent with the global methylation patterns, we found that a much higher proportion of DMFs were hypermethylated in the melanoma cell lines. Compared to 53T and 60T, 88% and 83% of the DMFs were significantly hypermethylated in the cell lines, respectively (Figure 3B,C, Supplementary Figure S2). The methylation of these DMFs increased from normal melanocyte cell lines, to melanoma tissue, to derived cell lines (median methylation of the DMF regions in normal melanocyte, metastatic tumours and their corresponding cell lines = 0.05, 0.37 and 0.78, respectively; Figure 3E). Of the 783 common DMFs, 737 (94.1%) were hypermethylated in both cell lines, as compared to their corresponding melanoma tumour tissues. One DMF was hypomethylated in both cell lines and the remaining 45 (5.7%) common DMFs showed methylation changes that were in a different direction in cell line and tissue comparisons (Supplementary Table S7). Of the 737 DMFs showing consistent hypermethylation in the cell lines, 131 DMFs were within gene promoters (consisting of 105 genes). A further 408 DMFs (associated with 326 genes) were within the gene bodies (exons, introns or exon-intron boundaries). We also found that 18 of these genes showed hypermethylation in the cell lines in both promoter and gene body regions. Previously, in an array-based study (Illumina 27K methylation array), 549 genes were reported to show differential methylation due to long term cell culture of mesenchymal stromal cells [34] and overlap analysis identified 16 of these genes were also identified in our study. Further, we also utilised a previous RRBS study that reported 690 genes with cell culture-related DNA methylation changes compared to primary tissues [22] and we found that 41 of these genes were differentially methylated in our study. Although there was a difference in the tissue type analysed and the platform used for methylation analysis, and while the previous studies were not performed in matched tissues and derived cell lines, these genes might still provide strong candidates for alteration of methylation levels due to cell culture.

### 2.5. Differentially Methylated Fragments Shared between Tumour Tissues and Derivative Cell Lines Are Involved in Tissue Morphogenesis and Cellular Differentiation Processes, and Are Enriched for the H3K27me3 Histone Mark and PRC2 Complex Genes

**Figure 4:**
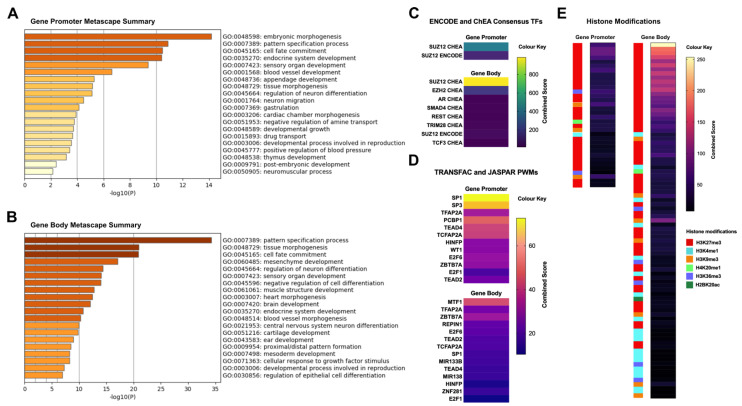
Analysis of differentially methylated fragments shared between tumour tissues and derived cell lines, with regard to gene enrichment and transcriptional function. (**A**,**B**) Enriched gene ontology terms relative to the shared DMFs that overlapped either gene promoters (−2 kb to +1 kb from the TSS) or gene bodies; the *x* axis represents −log10 of *p*-values. (**C**–**E**) Heatmaps of transcription factor (TF) targets (ENCODE/ChEA, TRANSFAC/JASPAR positional weight matrices) and histone modifications (Epigenomics Roadmap) significantly enriched (adjusted *p*-value < 0.01) in gene promoter and gene body DMFs.

To understand the possible functional roles of the genes hosting the shared differential methylation, we performed enrichment analysis on 105 common promoter methylation-associated genes and the 326 gene body methylation-associated genes. Gene ontology enrichment revealed an overrepresentation of genes associated with pattern specification, cell fate commitment, tissue morphogenesis and cellular differentiation related functions (Figure 4A,B, Supplementary Tables S8 and S9). Next, we utilised ENCODE Chip-Seq data and overlapped these genes with Chip-Seq peaks. We found that both promoter- and body-associated genes were predominantly enriched for H3K27me3 histone mark peaks (adjusted *p*-value < 0.01, Figure 4E, Supplementary Tables S10 and S11). Consistent with this finding, when we analysed the Chip-Seq and ChEA transcription factors, we found significant enrichment of PRC2 complex-related genes such as *SUZ12* and *EZH2* (Figure 4C, Supplementary Tables S10 and S11) for both promoter- and gene body-associated genes. These findings are consistent, as the H3K27me3 mark is a hallmark of PRC2 complex binding in a cell. We also assessed transcription factor (TF) binding and found significant enrichment of several common TFs such as TFAP2A, E2F6, SP1, E2F1, TEAD2 in promoter- and gene body methylation-associated genes (Figure 4D). These transcription factors have well established roles in development and in tumour progression [35,36,37].

## 3. Discussion

Here, we provide the first reduced representation bisulfite sequencing-based methylation maps of metastatic melanoma tumour tissues and their corresponding derivative cell lines, allowing assessment of the extent to which the cell line model represents their tumour tissue of origin DNA methylation patterns. Cancer cell lines are most valuable as in vitro models for cancer epigenetics research if their DNA methylation profile are in high concordance with the tumour tissues. We found that in both of our analysed pairs, the global methylation patterns are highly conserved between the tumour tissues and corresponding cell lines, and the cell line methylation is representative of their tumour tissue of origin. This observation corroborates previous epigenetic studies in melanoma, where identified methylation pattern in melanoma cell lines were validated in melanoma patient cohorts [33,38].

The pairs that we have analysed showed variation in their RRBS global methylomes (i.e, 60C and 60T were substantially more hypomethylated than the 53 sample pair). However, this is consistent with known inter-tumour heterogeneity of methylation levels. A recent whole genome bisulfite sequencing study which profiled nine cell lines from different tumours reported global methylation levels in those lines that ranged from 41.8% to 65.4% [25].

In this study, we identified 783 regions that showed common differential methylation between both the analysed pairs of cell lines compared to their tumour tissue of origin and 94.1% of these regions were hypermethylated in the cell lines. In a previous study, RRBS analysis was performed between embryonic stem cells (ES) and ES cells differentiated in vitro into neural precursor cells (NPC). The global methylome was significantly correlated between the ES cells and in vitro derived NPCs (rho = 0.81). However, a much higher number of CpGs (~8%), that were unmethylated in ES cells, became methylated in NPCs, and a relatively smaller proportion of CpGs (2%) that were methylated in ES cells, became unmethylated in NPCs [28]. Further, a large study that analysed specific regions in 24 different cell lines and 114 malignant samples, also reported extensive CpG island hypermethylation in cancer cell lines versus primary human malignancies (57% of the analysed fragments were methylated in cell lines but never methylated in malignant samples) [39]. Several other studies have provided evidence for region-specific DNA hypermethylation due to in vitro cell culture [40,41,42].

Previous studies have identified common hypermethylated loci in multiple cell lines, when compared to tumour tissue [39]. Here, we identified many differentially methylated loci (most of which were associated with hypermethylation), where methylation differences between the tumour tissue and the derivative cell line were shared in both of the cell lines, suggesting that hypermethylation events seen in cancer cell lines are likely to be associated with intrinsic properties of tumour cells grown in culture, rather than in their tumour tissue of origin. It is plausible that the hypermethylated loci or genes could be different for different tumour types and derivative cell lines. One explanation for this event could be that some genes need to become hypermethylated in order to alter expression of the associated transcription program required for these cell lines to maintain a selective growth advantage in vitro [43]. Another potential explanation is that tissue patterning or tissue morphogenesis genes are no longer required for cells to grow in 2D culture in vitro, and so those genes become progressively silenced when cells adapt to cell culture. These changes could be limited to cell culture and might not be tolerated for development in vivo, which is an important consideration for future epigenetic studies involving in vivo models. Future analysis involving perturbation of these cell line-specific genes could provide further insight into the role of these genes in cell growth in vitro. Other studies have shown locus-specific DNA methylation changes associated with culture-specific conditions, such as length of time in culture, passage number [34,44,45,46], and altered levels of sera in media [47,48,49]. Similar to our analyses here, these studies generally showed global methylation patterns were maintained throughout long-term culture, but some significant differences were observed at specific CpG sites. Indeed, it is well established that although DNA methylation patterns are influenced by environmental cues, they are relatively stable in somatic cells.

DNA methylation establishes and stabilises cellular phenotypes by maintaining gene expression states, which means that specific DNA methylation patterns are characteristic of particular cell types, lineages and phenotypes [4]. Although we aimed to use tissues with a high level of tumour cell purity, this phenomenon contributes to epigenetic heterogeneity within a tumour tissue microenvironment, comprising different cell types [50]. Similarly, cell lines also contain subpopulations of epigenetically and phenotypically distinct cells, which contributes to epigenetic heterogeneity [51,52]. The establishment of cancer cell lines leads to clonal selection, resulting in the existence of substrains that are likely due to clonal evolution during long-term culture. The diverging genomic and transcriptomic profiles of these substrains, which are intricately interwoven with the epigenome, can affect cancer cell malignancy and drug response [53]. Taking all these factors into consideration, it is likely that the potential epigenetic heterogeneity within the tumour tissues and their derivative cell lines had some impact on DNA methylation patterns. To fully evaluate this effect would require using methods such as single-cell lineage tracing [54,55] or bioinformatic deconvolution [56].

We discovered that the genes associated with the common site-specific methylation differences between cell lines and their corresponding tumour tissues are very strongly enriched for the repressive H3K27me3 mark. Polycomb Group (PcG) proteins including Polycomb Repressive Complexes, PRC1 and PRC2 are widely recognized to mediate gene silencing of developmental genes and also play a role in repressing genes in cancer [57,58]. One hallmark of the PcG domains is that they are marked by H3K27me3 histone, which is deposited by the catalytic component genes of the PRC2 core complex such as SUZ12, EZH1 and EZH2 families [58]. Consistent with our finding of H3K27me3 enrichment of the common differentially methylated genes, we also found highly significant enrichment of SUZ12 and EZH1 binding in these genes (determined by ChiP-Seq and ChEA data from multiple cell types). Together, we provide strong evidence that the cell line-specific hypermethylated genes are marked by PRC2 complex and H3K27me3.

It was reported that for a set of genes highly enriched with H3K27me3, CGI promoters were aberrantly hypermethylated in cancer cells but in not in normal cells [59]. Although many genes could be marked by H3K27me3, cell lines could gain promoter methylation in certain H3K27me3 genes in order to suppress them. This could potentially provide them with a growth or survival advantage, and it also suggests that methylation is a subsequent and causal event that can determine the fate of the expression of these genes, based on biological conditions (i.e., cell culture establishment). Further support in favour of this idea comes from a study profiling H3K27me3 in 19 human cell lines [60]. This analysis identified several high plasticity regions (HPRs) in the cell lines, which were enriched for the H3K27me3 mark and were associated with CpG island proximal or distal regions, and that these regions showed high variability across cell type. In addition, in a separate study, when the colon cancer cell line HT29 was compared with normal or tumour tissue samples, H3K4me3 showed similar profiles. However, the H3K27me3 mark exhibited significant differences in cell lines as compared to normal and colon tumour tissues [61]. A similar observation was also reported in breast cancer [62]. This combined evidence indicates that it is likely that cell lines use hypermethylation of these specific regions (such as high plasticity regions) enriched with H3K27me3 to gain a selective advantage for growth or survival in vitro. How PRC2 targets regions of the genome to be methylated, and the role and co-operation of H3K27me3, remain poorly understood in general [63], and warrant carefully designed mechanistic studies to understand their roles in different biological contexts.

We have analysed two pairs of tissues and their corresponding derivative cell lines, and it could be argued that this is a low sample number. However, such materials (i.e., tumour tissue and cell lines derived from the same tissue) are very rare, and to our knowledge, this study provides an RRBS map of tissues and matched derivative cell lines for the first time, for any tumour. Importantly, in both of our pairs independently, we observed consistent patterns of methylation, and overall conservation of global methylation, consistent with site-specific hypermethylation in the cell lines compared to the tissues. These results further indicate a common pattern of methylation in cell lines, as compared to tumour tissues in this study. A potential limitation is the absence of gene expression data for these samples. However, our hypothesis was that gene expression is a consequential event to genetic and epigenetic alterations, and we primarily wanted to investigate the nature of conservation and changes in DNA methylation, due to the establishment of cells in in vitro culture. Future studies with gene expression profiles of matched tissues and derivative cell lines will provide additional insight into the changes in expression program, which are due to establishment of cells in in vitro culture.

## 4. Materials and Methods

### 4.1. Ethics Statement

The New Zealand melanoma (NZM) tissue samples and their derivative cell lines used in this study were from surgical samples of two patients diagnosed with metastatic melanoma, coded as NZM53 and NZM60. For this paper, the NZM53 and NZM60 cell lines are designated 53C and 60C, respectively, to distinguish them from their tumour tissues of origin, 53T and 60T, respectively. The original tumour samples were obtained with written consent from each patient under the guidelines and specific approval of the Auckland Area Health Board Ethics Committee, New Zealand.

### 4.2. Molecular Classifications of Cell Lines

According to comprehensive molecular profiling of NZM cell lines [64], Sequenom Massarray analysis indicated that 53C and 60C each harbour an oncogenic mutation (TERT C250T and NRAS Q61H, respectively), but were negative for other hotspot mutations commonly found in melanoma (BRAF, PIK3CA, GNA11, CTNNB1, KRAS, PDGFRA, PTK2B, MET). Another study showed that 53C had a gene expression signature consistent with reduced invasive capacity [65]. We used Bis-SNP (RRID:SCR_005439) with default parameters [66] to detect single-nucleotide polymorphisms (SNPs) from the RRBS data. Our analysis of 7542 SNPs in common between 53T and 53C and 11615 SNPs in common between 60T and 60C showed that there was 99% and 75% identity, respectively, between the tissues and derivative cell lines. These analyses indicated clonality in terms of genetic mutation in the cell lines. Although this was an opportunistic use of the RRBS data, it is a sub-optimal method for definitive calling of SNPs. Although our findings were indicative, true SNP analysis requires a much higher coverage depth for accurate calling of SNPs. The ages of the patients at the time of the sample for NZM53 and NZM60 were 50 and 47, respectively. Immediately following tumour resection from the patients, the cell lines were established after approximately 3 months of culturing and passaging. Therefore, there will be no significant difference between the tumour and each corresponding cell line with respect to age-matching.

### 4.3. Tissue Sections

DNA was extracted from freshly cut sections of formalin-fixed paraffin-embedded (FFPE) melanoma tissue with the QIAamp DNA FFPE tissue kit, using xylene to dissolve the paraffin [67]. The two tissue DNA samples were labeled as 53T and 60T.

### 4.4. Cell Culture Establishment

Melanoma cells were isolated from pathologically-confirmed metastatic malignant melanomas and long-term cell cultures were established as previously described [68]. For this study, cells were revived and cultured for 72 h in Dulbecco’s modified Eagle medium (DMEM) containing 10% fetal calf serum (FCS) and labeled as 53C and 60C. Cell lines were checked for typical growth morphology, immunophenotype and in vitro differentiation potential [33,65].

### 4.5. RRBS Library Preparation and Sequencing

We used reduced representation bisulfite sequencing (RRBS) to map genome-wide DNA methylation as we have described previously [29,69,70,71]. Briefly, genomic DNA from each sample (53T, 53C, 60T and 60C) was digested with MspI enzyme followed by end-repair and ligation of Illumina TruSeq sequencing adaptors. Fragments were then size selected (40–220 bp) and bisulfite converted using the EZ DNA methylation direct kit prior to 16–18 rounds of PCR amplification. The quality and size distribution of the RRBS libraries were determined using an Agilent Bioanalyzer and quantified using a Qubit fluorometer. The libraries were sequenced on a single flow cell lane of an Illumina HiSeq 2000 sequencer (100 bp single-ended reads). Base-calling of the reads was performed with Illumina Real Time Analyzer (RTA) software.

### 4.6. DNA Methylation Data Analysis

As previously described, the sequenced RRBS reads were quality checked using FastQC (RRID:SCR_014583) and processed using bioinformatics tools developed in house [29,30]. The Bismark (RRID:SCR_005604) alignment tool [72] was used to map the processed sequence reads to the reference human genome (GRCh37), allowing only one mismatch in the seed. After filtering out low quality sequences, the median number of uniquely aligned reads for the RRBS libraries was 6.7 million. The median non-CpG DNA methylation was 1.95% (as measured by Bismark alignment), indicating effective bisulfite conversion and low levels of non-CpG methylation. For comparative purposes, we also utilised previously described RRBS data from three normal melanocyte cell lines (the SV40-transformed line Mel-ST and non-transformed lines, HEMn-LP and HEMa-LP) and 12 metastatic melanoma cell lines (CM145-post, CM145-pre, CM150-post, CM142-post, CM138, CM143-pre, CM143-post, MEL-RMU, NZM9, NZM22, NZM40 and NZM42) [32,33,73].

### 4.7. Differential Methylation Analysis

We used our in-house Differential Methylation Analysis Package (DMAP) to identify RRBS fragments that were differentially methylated between the tissues and their derived cell lines [30]. Briefly, we applied a pairwise Fisher’s exact test on 53T vs. 53C and 60T vs. 60C, respectively. For the analysis of RRBS methylomes, we have employed a MspI fragment-based analysis approach. The fragment-based analysis approach for RRBS has been well described previously [30,31,74,75]. We restricted our analysis to fragments with at least two CpG sites covered by 10 or more sequenced reads. To filter for significantly differentially methylated fragments (DMFs), we applied Bonferroni correction (at a significance level of 0.01) to correct for multiple comparison testing and we obtained fragments with ≥25% difference in methylation between 53T vs. 53C and 60T vs. 60C, respectively. Gene information for all fragments, including location of gene promoters, introns and exons, was derived using the *identgeneloc* program of the DMAP tool [30]. We used BEDTools (RRID:SCR_006646) to overlap the methylation data with several genomic elements and regulatory features: enhancers (GeneHancer, https://genecards.weizmann.ac.il/geneloc/index.shtml, accessed on 7 April 2017), super-enhancers (H3K27Ac ChIP-seq data of A375 cells, GSE99835), CTCF binding sites (CTCF ChIP-seq data of A375 cells, GSE128346) and specific repeat classes (RepeatMasker, https://www.repeatmasker.org, accessed on 25 September 2018).

### 4.8. Functional Enrichment Analyses of the Genomic Features

We used Metascape (RRID:SCR_016620) [76] and Enrichr (RRID:SCR_001575) [77] to perform functional enrichment analyses on differentially methylated gene sets. The gene sets were comprised of either gene promoters (−2 kb to +1 kb from the TSS, n = 105) or gene bodies (n = 326) that overlapped DMFs common to both the 53T vs. 53C and 60T vs. 60C analyses. For Metascape analysis, gene sets were queried against the background of all protein-coding human genes overlapping commonly analysed RRBS fragments. For Enrichr analysis, we used: (1) the ENCODE/ChEA Consensus database, which incorporates the Encyclopedia of DNA Elements (ENCODE) project and Chip-X Enrichment Analysis (ChEA) datasets; (2) positional weight matrices from TRANSFAC and JASPAR databases; and (3) Epigenomics Roadmap histone modification ChIP-seq data.

## 5. Conclusions

In conclusion, our sequencing-based genome-scale analysis demonstrates a global conservation of DNA methylation patterns following the establishment of in vitro cell cultures, which had been derived from matching human metastatic melanoma tissues, and therefore provides supporting evidence in favour of using cell lines as in vitro models for methylation studies in cancer epigenetic research and drug discovery. Our results also provide an impetus for conducting similar studies in other cancer types to assess methylation patterns. We also demonstrate region-specific epigenomic differences between the cell lines and the corresponding matching tumour tissues, and reveal novel genomic features of these regions, thus providing a valuable resource for cancer methylome studies to reduce false-positive candidates from the epigenetic effects of cell culture. This resource will allow better interpretation of cancer epigenetics studies in future.

## Data Availability

All data generated in this study have been deposited in NCBI under the submission number GSE159741.

## Supplementary Materials

The following are available online at https://www.mdpi.com/article/10.3390/cancers13092123/s1, Figure S1: Proportion of common analysed fragments overlapping genomic elements in melanoma tissues and derived cell lines, Figure S2: Frequency distribution of differentially methylated fragments overlapping genomic elements in melanoma tissues and derived cell lines, Table S1: Sequencing and mapping details of the four RRBS libraries, Table S2: Details of analysed fragments and CpG sites in tissue and cell lines, Table S3: Details of global methylation correlation in tissue and cell lines, Table S4: Details of global methylation distribution in tissue and cell lines, Table S5: Differentially methylated fragments between 53C and 53T, Table S6: Differentially methylated fragments between 60C and 60T, Table S7: Common differentially methylated fragments, Table S8: Metascape GO enrichment of common gene promoter DMFs, Table S9: Metascape GO enrichment of common gene body DMFs, Table S10: Enrichr analysis of common gene promoter DMFs (adjusted *p*-value < 0.01), Table S11: Enrichr analysis of common gene body DMFs (adjusted *p*-value < 0.01).
