# Supplementary material for: Comparison of Global DNA Methylation Patterns in Human Melanoma Tissues and Their Derivative Cell Lines
Source: Cancers (Basel). 2021 Apr 28;13(9):2123. doi: 10.3390/cancers13092123 (PMC8124222; doi:10.3390/cancers13092123)
Supplement: Supplementary file 1 [file cancers-13-02123-s001.zip › cancers-1115462-supplementary figures.pdf]

cancers

MDPI

Supplementary Materials:

Comparison of Global DNA Methylation Patterns in Human Melanoma Tissues and Their Derivative Cell Lines

Euan J Rodger, Suzan N Almomani, Jackie L Ludgate, Peter A Stockwell, Bruce C Baguley, Michael R Eccles and Aniruddha Chatterjee

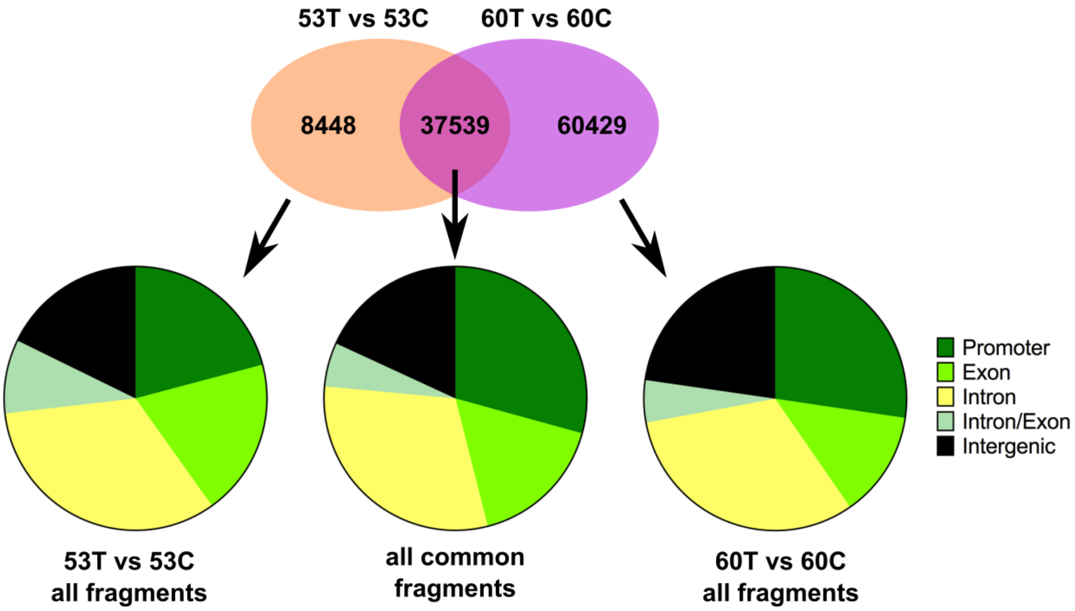

Figure S1. Proportion of common analysed fragments overlapping genomic elements in melanoma tissues and derived cell lines. The Venn diagram shows the number RRBS fragments with high quality information (fragments having 10 or more reads at ≥2 CpG sites) in each comparison and the 37539 that were in common. The pie charts show the proportion of common analysed RRBS fragments overlapping gene promoters (−2 kb to +1 kb from the TSS), exons, introns, intron/exon junctions and intergenic elements (>2 kb upstream from the nearest TSS).

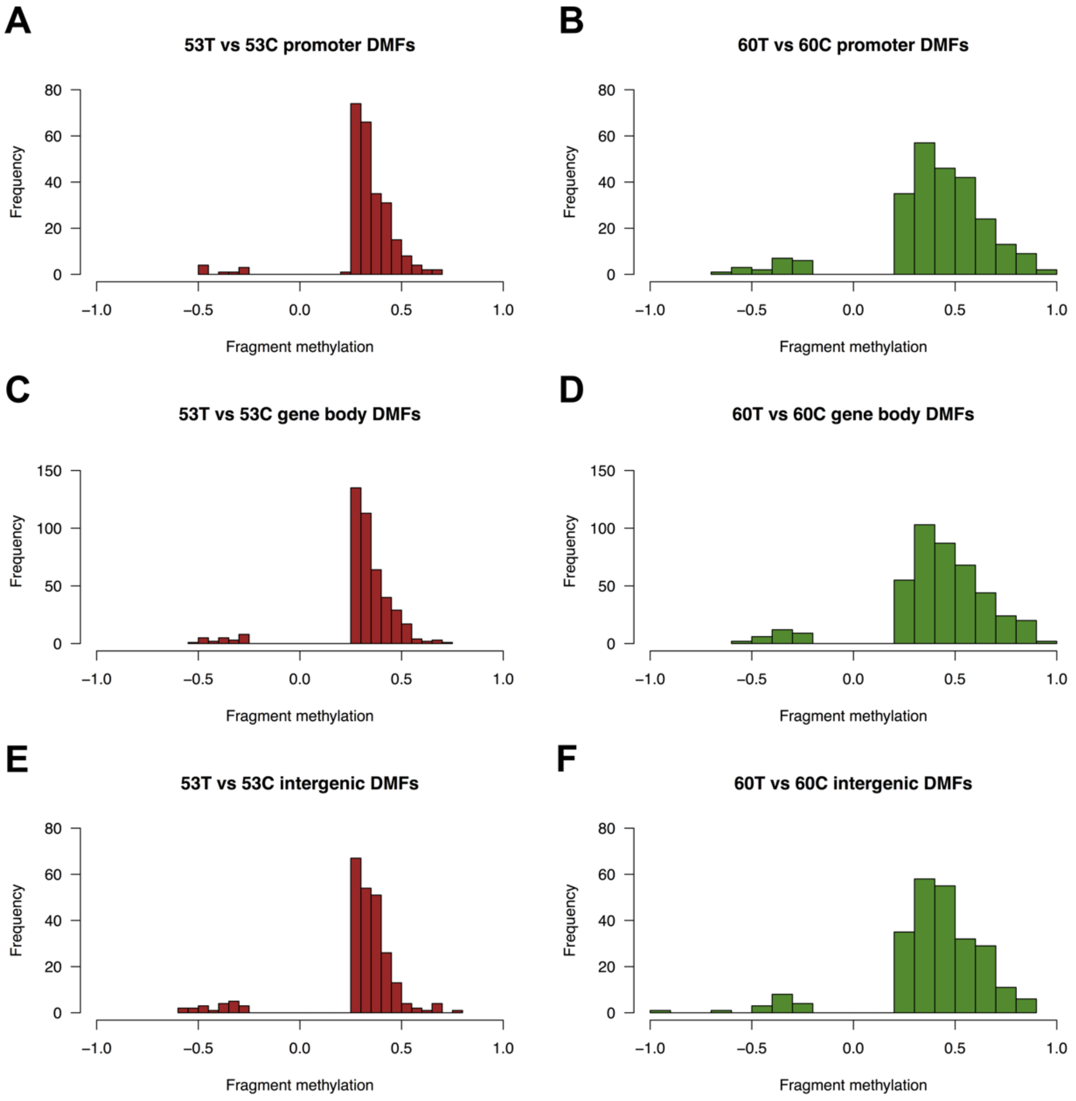

Figures S2. Frequency distribution of differentially methylated fragments overlapping genomic elements in melanoma tissues and derived cell lines. Frequency histograms of significantly differentially methylated fragments (DMFs) overlapping gene promoters (-2 kb to +1 kb from the TSS) in the 53T vs 53C (**A**) and 60T vs 60C comparisons (**B**), of DMFs overlapping gene bodies in the 53T vs 53C (**C**) and 60T vs 60C comparisons (**D**), and of DMFs overlapping intergenic regions in the 53T vs 53C (**E**) and 60T vs 60C comparisons (**F**).
